# Hematologic biomarkers in childhood cataracts

**Published:** 2011-04-24

**Authors:** O. Wussuki-Lior, A. Abu-Horowitz, I. Netzer, Z. Almer, Y. Morad, Y. Goldich, V. Yahalom, El. Pras, Er. Pras

**Affiliations:** 1 Department of Ophthalmology, Assaf Harofeh Medical Center, Zerifin, Israel; 2 Sheba Medical Center, Danek Gartener Institute of Human Genetics, Tel Hashomer, Israel; 3 Sackler School of Medicine, Tel Aviv University, Tel Aviv, Israel; 4 National Blood Group Reference Laboratory (NBGRL), Magen David Adom (MDA) – National Blood Services, Ramat Gan, Israel

## Abstract

**Purpose:**

To date, more than thirty nine genetic loci have been associated with congenital cataracts. Despite this progress, current diagnostic techniques are insufficient for unraveling the underlying genetic defect in sporadic patients and small families. In the present manuscript we demonstrate the contribution of routine laboratory tests in the search for genetic defects of childhood cataracts.

**Methods:**

Two families with congenital cataracts and hematologic findings that included hyperferritinemia and the “ii” blood type underwent detailed ophthalmologic and clinical examinations. Mutation analysis of the ferritin light chain (*FTL*) and glucosaminyl (N-acetyl) transferase 2, I-branching enzyme (*GCNT2*) genes was performed in the two families, respectively.

**Results:**

In the family with the “ii” blood group we found a novel *GCNT2* mutation c.G935A (p.G312D) in the cataract patients, while in the family with hyperferritinemia cataract syndrome we identified a G→C heterozygous mutation at position +32 of *FTL*.

**Conclusions:**

Hematologic biomarkers may simplify the search for the underlying molecular defect in families with congenital cataract.

## Introduction

Congenital cataract encompasses many different diseases with distinct causes and diverse biologic pathways that result in crystalline lens opacities. While senile cataract is considered a common treatable disorder of the elderly, congenital cataract is particularly serious because it has the potential of inhibiting visual development, and may result in permanent blindness. The frequency of congenital cataract is estimated at 1–6 per 10,000 live births, and up to one third of them are inherited [1]. They vary markedly in severity and morphology, affecting the nuclear, cortical, polar or subcapsular parts of the lens, or in severe cases the entire lens [2]. The phenotype by itself is not a good predictor of the underlying gene or mutation since identical cataracts can result from mutations at different genetic loci, and may have different inheritance patterns. Contrarily, various cataract types can be found in a single large family [3]. Usually, congenital cataracts occur in an isolated fashion affecting the lens alone or in conjugation with other ocular anomalies such as microphthalmia, aniridia, and retinal degenerations. They may also be associated with myriad systemic conditions including chromosomal abnormalities; craniofacial, mandibulofacial, and skeletal syndromes; metabolic disorders; congenital infections; dermatologic, central nervous system (CNS), musculoskeletal, or renal disease [4]. More than 39 genetic loci for cataract have been mapped, and in more than twenty-five of them specific genes have been identified (Cat-Map*)*. These tools have been very successful in determining the underlying genetic defect in large pedigrees and in sporadic patients whenever cataract manifests as one component of a multisystem syndrome as in Lowe syndrome or neurofibromatosis type-2 [3-5]. However, in sporadic patients and small families with non-syndromic congenital cataract it is almost an impossible task. Nevertheless, there are instances where the distinction between syndromic cataracts and isolated ones is less evident, and congenital cataracts are accompanied by occult abnormalities in other organs.

In the present study we describe two small families of congenital cataract and abnormal blood tests which suggested their underlying pathology. In one of the families the association of high serum ferritin levels with cataract guided us to search for mutations in the ferritin light chain (*FTL*) gene, while the finding of “ii” blood type in affected members of the second cataract family lead us to look for mutations in the glucosaminyl (N-acetyl) transferase 2, I-branching enzyme (*GCNT2*) gene.

## Methods

**Figure 1:**
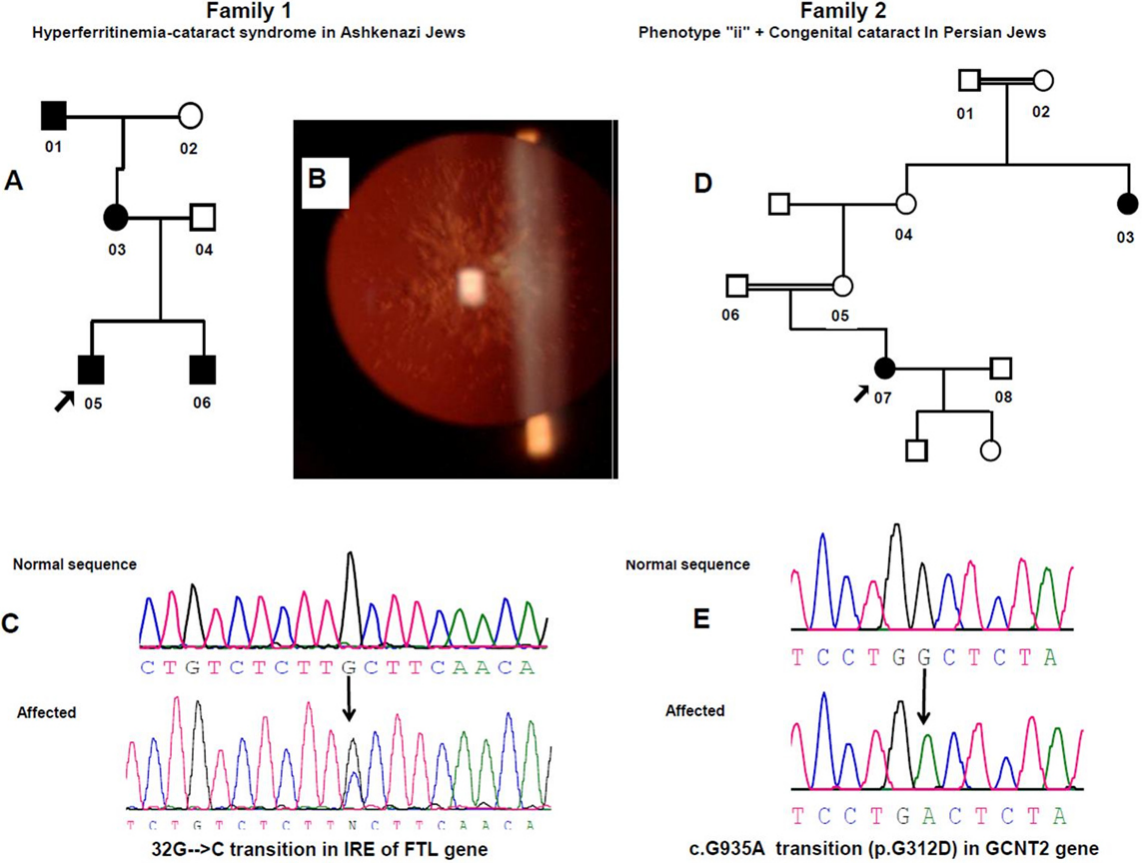
Clinical features and genetic analysis. **A**: Ashkenazi Jewish family pedigree affected by Hereditary Hyperferritinemia Cataract Syndrome. **B**: Slit-lamp retroilluminative view of the lens discloses nuclear cataract with prominent Y sutures. **C**: DNA sequence analysis of the IRE (iron responsive element) part of *FTL* (ferritin light chain). A heterozygous G→C change in the 5`-untranslated region (5`-UTR) at position +32 from transcription start site (c. −168G→C), is indicated by black arrow. **D**: Persian Jewish family pedigree affected by congenital cataract (no photograph available) and phenotype ii. **E**: DNA sequence analysis showing a homozygous G → A transition (indicated by black arrow) at cDNA position 935, resulting in a change of Glycine to Aspartic acid (p.G312D) of all three isoforms of *GCNT2*.

The study protocol adhered to the provisions of the Declaration of Helsinki and informed consent was obtained from the participants. The two families were recruited at the Genetic Eye Clinic, Assaf Harofeh Medical Center, Zerifin, Israel (Figure 1A,D). Family members underwent a detailed ophthalmologic examination, which included slit lamp biomicroscopy with photography of the cataract lenses (when possible). Heparinized blood was obtained for genomic DNA isolation, blood typing, serum ferritin and iron levels, and total iron binding capacity.

### I/i blood group and serum ferritin level testings

I/i phenotype was tested at the Israeli National Blood Group Reference Laboratory (NBGRL) at Magen David Adom Blood Services in Israel. Testing was performed by conventional (tube) methods [6], using anti-I and anti-i from Serum Cells and Rare Fluids (SCARF) and anti-i from our in-house anti-sera collection. Cord red blood cells were used as a positive control for i and negative control for I. Adult red blood cells were the positive control for I and negative control for i. Serum ferritin levels were determined with the electrochemiluminesence immunoassay “ECLIA”method (Elecsys and cobas e analyzers) [7].

### *FTL* and *GCNT2* mutation screening

The Iron Responsive element (IRE) at the 5-'UTR region of *FTL* and all exons and exon-intron boundaries of the *GNTC2* gene were amplified from genomic DNA using specific primer pairs (Table 1), and sequenced with BigDye Terminator cycle sequence kit v3.1 (Applied Biosystems, Inc. ABI, Foster City, CA) according to manufacturer's instructions. One hundred chromosomes of Ashkenazi Jewish and Persian Jewish origins without any known ocular diseases were used as controls.

**Table 1:** Primers for PCR amplification of *GCNT2*, and the Iron Responsive Element (IRE) of the *FTL* gene.

**Gene**	**Annealing temperature**	**Fw primer**	**Rv primer**	**Product size (bp)**
*GCNT2* exon1A	60 ºC	5’-TGTAGACACAGGTTGCAGGTTAGCA-3’	5’- GCAGGTAGCTTCATCAAGGGTA -3’	222 bp
	55 ºC	5’-TAGCAGAAGCCTGTCATCAG-3’	5’- CCTTCAGATACTGAACTATTTC -3’	491 bp
	55 ºC	5’-AACACCTGCGGGCAAGACTT-3’	5’-CTTTTGTCCTGTGAACAGAGCGGTT-3’	570 bp
*GCNT2* exon1B	55 ºC	5’-AGACTTACAGATTTTGACGGT-3’	5’-TAGATATTTTGGGGCATGTA-3’	414 bp
	57 ºC	5’-CCATCATCACTTTGACACCT-3’	5’-CTTATCACATAGGAAAGCTCT-3’	429 bp
	55 ºC	5’-CTCATGCAATTGGACGGACT-3’	5’-GGGTGAGAACTATATATGTTCCAGTT-3’	380 bp
*GCNT2* exon1C	55 ºC	5’-GCAAATTCAACCTCTCACACCGATC-3’	5’-GGGGCATATAGATAGCCCTAA-3’	437 bp
	55 ºC	5’-TGTCATGGTCATCCATAAGG-3’	5’-CTTGGTGGACATATTTAGTT-3’	407 bp
	55 ºC	5’-AGGATTTAAAGGGAAAAATATC-3’	5’-TGAGTCAGTTCTCTAGGCGAGCAG-3’	374 bp
*GCNT2* exon2	57 ºC	5’-CTGAAGTGGAGAAACCCTGGCTTA-3’	5’-AACCCTGGATTCCACAGCTACCTT-3’	514 bp
*GCNT2* exon3	55 ºC	5’-AGTTGTAGTTAGTCGGAGAGTACCT-3’	5’-TATAATTACGTAGCCAGGTCCTGAA-3’	430 bp
IRE of *FTL*	58 °C	5’-GGCTGTTAGTGCTCCCATAA-3’	5’-GATCTGTTCCGTCCAAACAC-3’	383 bp

## Results

### Family 1

This is a relatively small three generation Ashkenazi Jewish family emigrated from Romania to Israel 40 years ago (Figure 1A). The index case (individual 05), a six-year-old child was diagnosed as having bilateral nuclear cataracts (Figure 1B), with visual acuity of 6/12 in both eyes. A detailed ophthalmologic examination performed on his eighteen months old brother (individual 06) revealed mild sutural cataract. Slit-lamp biomicroscopy demonstrated a nuclear cataract with prominent Y sutures in both children. After discussing the option of surgical treatment with the parents a decision for conservative follow-up was made.

The family history revealed that the mother (individual 03) had visual disturbances since childhood and underwent cataract extraction at the age of 41. The maternal grandfather (individual 01) had bilateral decreased vision since childhood and had cataract surgery at the age of 40.

Since cataract extraction, both the mother (individual 03) and her father (individual 01) were both pseudophakic. Otherwise no other ophthalmic pathology was found. At the age of 45, as an incidental finding individual 03 was found to have elevated ferritin levels in the absence of iron overload, ranging between 1,393 and 1,621 ng/ml (normal: 20–167 ng/ml). Serum ferritin level in individual 05 was 1,161 ng/ml, and 1,300 ng/ml in individual 01.

The tenfold increase in L-ferritin levels in association with early onset nuclear cataract was consistent with the diagnosis of Hereditary Hyperferritinemia Cataract Syndrome (HHCS; OMIM 600886). Sequencing *FTL* in the 4 affected family members (individuals 01, 03, 05, and 06) showed a heterozygous change G→C (Figure 1C) at position 32 from the transcription start site (c. −168G→C). This sequence variation occurred in the iron responsive element (IRE) located at the 5′-UTR of *FTL*. The change was not seen in the unaffected family members (individuals 04 and 02) nor was it found in 200 ethnically matched control chromosomes.

### Family 2

The index patient (individual 07) a daughter of first cousin parents of Persian Jewish decent was noticed to suffer from congenital cataract soon after birth. Family history revealed a great maternal aunt (individual 03), born to consanguineous parents, who also had congenital cataract (Figure 1D). By history both had bilateral leukocoria (white pupil) evident during early infancy and opaque lenses that prevented ophthalmoscope retinal examination before lens extraction. Ophthalmic examination of the index case and her great aunt (individuals 07 and 03) revealed bilateral blindness with very low visual acuities ranging from hand motions to counting fingers before eyes. Both had nystagmous, severe amblyopia, and aphakia. No pathology or photography of the lens was available. No ocular abnormalities were found in the parents and unaffected sibling. Blood typing performed on the index case (individual 07) before gynecological surgery revealed that she was homozygous for the ii blood group. Her great aunt (individual 03) was found to have the same ii blood group. Sequencing the three different *GCNT2* isoforms revealed a homozygous G→A substitution at position 935 of the cDNA (c.G935A), resulting in a change of an evolutionary conserved Glycine to Aspartic acid (p.G312D) in all three isoforms, *GCNT2A*, -*B*, and -*C* (Figure 1E). This change was not detected in 200 Persian Jewish control chromosomes.

## Discussion

The discovery of a broad variety of genes associate with congenital cataracts hurdles the search for the underlying causative mutation especially in sporadic patients and families too small for linkage studies. In the present study unraveling the underlying mutations in two congenital cataract families was relatively simple thanks to associated blood findings that focused the search to a single gene.

The first family was diagnosed with Hereditary Hyperferritinemia Cataract Syndrome (HHCS) due to a single nucleotide change (c. −168G→C) in the IRE of L-Ferritin mRNA, identified in the heterozygous state in all affected members. This substitution occurs in the highly conserved three-nucleotide bulge structure (positions 31–33) of *FTL* promoter (IRE) that is considered a mutation “hot-spot,” and many of the HHCS families described to this date carry mutations at the same nucleotide position (32G→U and 32G→A) [8-12]. This position 32G has been previously demonstrated to have a pivotal role in the regulation of *FTL* mRNA [13,14], resulting in upregulation of L-ferritin in the serum and body tissues [15,16]. Both of the affected brothers were examined at birth under the neonatal screening protocol implemented in Israel, which includes examination of the retinal red reflex. Both were found normal, supporting the hypothesis that the cataract in HHCS is not congenital, but develops later in life. These findings are in line with previous reports that some children with high serum ferritin levels and the same mutation as their affected relatives with cataract did not have cataract. Francesca et al. have suggested that the opacities may be the outcome of age-related ferritin accumulation in the lens [17]. Interestingly, we did find opacities in an 18 months old child from this family, one of the youngest patients described with HHCS cataract. Thus it is evident that other factors, environmental or genetic play a role in the timing of HHCS cataract development.

The second family of Persian Jewish origin had a different underlying disorder. Two members of this consanguineous family were found homozygotes for a novel mutation in *GCNT2*. This gene encodes for a specific transferase, I-branching β-1,6-N-acetylglucoseaminyltransferase (I β 6GlcNAcT) which is essential for the conversion of i into I antigenic structure on various cell types. Previous studies of the human I locus, located on chromosome 6p, revealed that *GCNT2* has 3 splicing variants, A, B, and C, which differ at exon 1 but have identical exon 2 and 3 coding regions, and are expressed differentially in specific tissues. Mutation events that occur in the specific exon 1 region of *GCNT2* may lead to a defect in one isoform of the GCNT2 enzyme and i phenotype in certain cell types, whereas those that occur in the common exon 2 to 3 region result in i phenotype as well as congenital cataract, due to the elimination of the activity of all three isoforms of the GCNT2 enzyme [18]. In agreement with the above, the mutation found in our family occurs in the second exon and therefore results in the i blood type as well as congenital cataract.

The two families described in this report highlight the need to rule out for systemic disorders before embarking a molecular search for mutations. Other examples where early onset cataract is the major manifestation of an underlying systemic disorder include renal glucosuria due to solute carrier family 16, member 12 (monocarboxylic acid transporter 12; *SLC16A12*) mutations [19], and lactose intolerance due to galactokinase 1 (*GALK1*) mutations [20]. We therefore propose to check blood glucose and ferritin levels, urine glucose and the i/I blood-type, as the first step of evaluation in such cases.
